# Unveiling the Antiviral Potential of Minocycline: Modulation of Nuclear Export of Viral Ribonuclear Proteins during Influenza Virus Infection

**DOI:** 10.3390/v16081317

**Published:** 2024-08-18

**Authors:** Priyanka Saha, Ritubrita Saha, Ratul Datta Chaudhuri, Rakesh Sarkar, Mehuli Sarkar, Hemanta Koley, Mamta Chawla-Sarkar

**Affiliations:** 1 Division of Virology, ICMR-National Institute of Cholera and Enteric Diseases, Kolkata 700010, India; 2 Division of Bacteriology, ICMR-National Institute of Cholera and Enteric Diseases, Kolkata 700010, India

**Keywords:** A/H1N1, minocycline, viral ribonucleoprotein, antiviral, ERK

## Abstract

Influenza A virus (IAV) poses a global threat worldwide causing pandemics, epidemics, and seasonal outbreaks. Annual modification of vaccines is costly due to continual shifts in circulating genotypes, leading to inadequate coverage in low- and middle-income countries like India. Additionally, IAVs are evolving resistance to approved antivirals, necessitating a search for alternative treatments. In this study, the antiviral role of the FDA-approved antibiotic minocycline against IAV strains was evaluated in vitro and in vivo by quantifying viral gene expression by qRT-PCR, viral protein levels by Western blotting, and viral titers. Our findings demonstrate that minocycline at a non-toxic dose effectively inhibits IAV replication, regardless of viral strain or cell line. Its antiviral mechanism operates independently of interferon signaling by targeting the MEK/ERK signaling pathway, which is crucial for the export of viral ribonucleoproteins (vRNPs). Minocycline prevents the assembly and release of infectious viral particles by causing the accumulation of vRNPs within the nucleus. Moreover, minocycline also inhibits IAV-induced late-stage apoptosis, further suppressing viral propagation. The antiviral activity of minocycline against IAVs could offer a promising solution amidst the challenges posed by influenza and the limitations of current treatments.

## 1. Introduction

Infants, elderly, immunocompromised, and chronically ill individuals are vulnerable to bacterial and viral respiratory infections including influenza viruses, notably due to their inability to curb the associated complications [1]. The last century has witnessed many outbreaks and pandemics starting with the 1918 Spanish flu caused by Influenza A/H1N1 virus, which claimed nearly 40 million lives globally [2]. Influenza A virus (IAV) is an RNA virus having eight negative-sense single-stranded RNA segments coding for 11 proteins. Each viral RNA segment associates with the nucleoprotein (NP) and the RNA-dependent RNA-polymerase, known as RdRp, that binds to the RNA molecule to form the viral ribonucleoprotein complex (vRNP). The life cycle of IAVs entails entry of the virus into the host, import of vRNPs into the nucleus, replication and transcription of the vRNAs, release of vRNPs into the cytosol, and the assembly and release of progeny virions [3,4]. Bidirectional transport of vRNAs is essential during viral replication. Nuclear export of vRNPs at the late stage of infection is initiated by the envelope protein hemagglutinin [5].

Influenza viruses are considered to be a serious threat from a public health perspective due to their high genetic diversity and capacity for genetic reassortment. Vaccines and antiviral drugs have been used in the prevention, control, and long-term management of viral epidemics. Nevertheless, the accumulation of point mutations and reassortment of gene segments result in rendering the existing therapy and vaccine less effective over a period of time. This underscores the necessity of global surveillance of circulating influenza strains in the world for continuous upgradation of vaccines [6,7,8,9,10,11,12,13]. Presently, two classes of drugs are employed to treat influenza: Neuraminidase (NA) inhibitors such as oseltamivir, peramivir, and zanamivir and Matrix protein 2 (M2) ion channel blockers like amantadine and rimantadine [14,15]. Resistance to M2 inhibitors has developed due to point mutations, namely L26F or S31N, while H274Y mutation in NA lowers its susceptibility to oseltamivir [16,17]. To overcome this, the focus has shifted towards searching for new antivirals that target host determinants that aid in virus replication.

Minocycline (7-dimethylamino-6-dimethyl-6-deoxytetracycline), a second-generation tetracycline analog, has favorable pharmacokinetics and efficient penetration of the blood–brain barrier [18]. It is generally well tolerated and prolonged use has been associated with few adverse effects [19]. Beyond its anti-inflammatory, anti-apoptotic, neuroprotective, and immune-modulating properties, minocycline also has shown antiviral potential [20,21,22]. Initially recognized for its activity against Human Immunodeficiency Virus (HIV), it has also shown efficacy against various infectious viruses such as Japanese encephalitis virus (JEV), Sindbis, Dengue virus (DENV), Respiratory Syncytial virus (RSV), and Reoviruses [23,24,25,26,27,28,29,30,31,32]. The present study establishes that the FDA-approved antibiotic minocycline inhibits influenza viruses by hampering the assembly and release of progeny virions via impeding both ERK-mediated nuclear export of vRNPs and virus-induced apoptosis.

## 2. Materials and Methods

### 2.1. Cell Culture and Virus Infection

Madin–Darby canine kidney (MDCK) and African green monkey fibroblast (Vero) cells were maintained in minimal essential medium (MEM) while human lung carcinoma (A549) cells and human embryonic kidney (HEK293T) cells were maintained in Dulbecco’s modified Eagle’s medium (DMEM) supplemented with 10% fetal bovine serum (FBS) and 1% antibiotic–antimycotic (penicillin and streptomycin–amphotericin B) at 37 °C with 5% CO_2_. MDCK, Vero, A549, and HEK293T cell lines were purchased from ATCC (CC-4, CRL-1586, CCL-185, and CRL-3216). MDCK, Vero, and A549 cells are adherent epithelial cell lines permissive to influenza virus infection [33,34,35,36]. The cells were infected with pandemic strain A/California/04/2009 (H1N1)pdm09 [NR-13658] {IAV/CAL}, Influenza A H3N2 strain (gifted by ICMR-NIV, Pune), and cell culture adapted virus strain A/Puerto Rico/8/1934 (H1N1) {IAV/PR8}. Viral titers of the cultured viruses were measured in terms of PFU/mL after 3 days post-infection using the plaque assay while the presence of the viruses was estimated in terms of HA titer using the hemagglutination assay. For in vitro infection, the cells were adsorbed with TPCK-treated trypsin-activated IAV strains for 1 h at 37 °C, with agitation after every 20 min. Following washing with PBS, infection was continued in a serum-free medium. The time of virus removal after adsorption was considered as 0 h post-infection (hpi). In the dose–response study, treatment of minocycline and ribavirin was carried out at 0 hpi. The cells were treated with ERK inhibitor PD98059 (30 µM) at 0 hpi. For all experiments, cells were infected at an MOI of 1.00.

### 2.2. IAV Infection In Vivo and Ethics Statement

Female BALB/c mice aged 4–6 weeks (obtained from an institutional animal facility) were orally administered with minocycline (5, 15, and 30 mg/kg/day) for 5 days to assess their toxicity. Survivability, body weight changes, and histological alterations in vital organs—lung, kidney, heart, and liver—were monitored. A non-toxic dose of minocycline (30 mg/kg/day) was administered from day 2 to day 15 after intranasal inoculation with IAV/PR8 (4 × 50% mouse lethal dose) and body weights were measured. Another group was infected with IAV/PR8 and treated with minocycline (30 mg/kg/day), DMSO (vehicle control), or ribavirin (70 mg/kg/day) from day 2 to day 4. On day 5, mice were sacrificed, and viral titers in the lung homogenates were determined by plaque or HA assay. Expression of viral protein NS1 and RNA (M1 gene) in lung homogenates was quantified by Western blotting and quantitative real-time PCR, respectively. Lung samples were fixed, sectioned, and stained with hematoxylin and eosin (H&E). Images were captured using an EVOS^TM^ XL Core microscope (magnification, 10× & 40×; Thermo Fisher Scientific, Waltham, MA, USA). Each experimental condition involved five mice per group. All the experiments were performed according to national regulations and approved by the institutional animal ethics committee (PRO/200/June 2023-26).

### 2.3. Reagents and Antibody

Minocycline HCl purchased from Apexbio (B1791) was used as 10 mM stock in DMSO (Dimethyl Sulfoxide). MEK/ERK pathway inhibitor PD98059 (M2865-17A.1500) was purchased from Biomol. Staurosporine (S5921), PMA (phorbol 12-myristate 13-acetate) (P1585), ribavirin (36791-04-5), and Interferon α-2β (H6166) were obtained from Sigma-Aldrich. Antibodies against nucleoprotein [NP] (ab128193) and non-structural protein 1 [NS1] (PA5-32243) were obtained from Abcam and Invitrogen, respectively. Antibodies against phospho-Janus kinase 1 [p-JAK1] (3331S), Janus kinase 1 [JAK1] (3332S), p-Signal Transducer and Activator of Transcription 1 [p-STAT1] (9171S), STAT1 (9172S), bax (5023), caspase-3 (9662), cleaved caspase-3 (9661), cleaved caspase-9 (9505), and Extracellular Signal-Regulated Kinase 1/2 [ERK1/2] (9102) were obtained from Cell Signaling Technology. Antibody against phospho-ERK1/2 [p-ERK1/2] (pT202/Y204) (612358) was purchased from BD Biosciences. Antibody β-actin (sc-47778) was purchased from Santacruz Biotechnology.

### 2.4. Quantitative Real-Time Reverse Transcription-PCR (qRT-PCR)

Modulation in viral mRNA transcripts within 6, 12, 24, 48, and 72 h of infection was analyzed. Total cellular RNA was extracted using TRIzol reagent (Invitrogen) according to the manufacturer’s instructions. cDNA was prepared from 500 ng of RNA using the Superscript II reverse transcriptase (Invitrogen) with random hexamer primers. Real-time PCR reactions (50 °C for 3 min, 95 °C for 2 min, followed by 40 cycles of 95 °C for 3 s and 60 °C for 30 s and followed by 65 °C for 5 s and 95 °C for 50 s) were performed in triplicate using PowerUP SYBR Green (Applied Biosystems). Primers specific for M1 and 18s rRNA were used for corresponding gene transcripts. The relative gene expressions were normalized to 18s rRNA using the following formula: 2^−ΔΔCT^ (ΔΔCT = ΔCT _Sample_ − ΔCT _Untreated control_).

### 2.5. Western Blot Analysis

After experimentation, the cells were washed with prechilled PBS and lysed in 1X cell lysis buffer under ice-cold conditions. Protein concentration was measured using Bradford reagent (Sigma-Aldrich, USA). Whole-cell lysates, cytosolic, and nuclear fractions were mixed with 6X Laemmli buffer (protein sample buffer) and boiled for 15 min [37]. Samples were run on SDS-PAGE, transferred onto a polyvinylidene fluoride (PVDF) membrane, and immunoblotted with specific antibodies as described previously [38]. These primary antibodies were detected using a secondary antibody conjugated with horseradish peroxidase (HRP) (ThermoFisher Scientific™, USA) and developed using a chemiluminescent substrate (Millipore) in the ChemiDoc Imaging System (Bio-Rad, Hercules, CA, USA). 

### 2.6. Immunofluorescence Microscopy

MDCK cells grown on glass coverslips (30–50% confluency) were either treated with minocycline or only DMSO and/or infected with the IAV/PR8 strain. IAV/PR8-infected cells treated with MEK/ERK inhibitor PD98059 (30 µM) served as the positive control. The cells were fixed in 4% (*w*/*v*) paraformaldehyde for 20 min, followed by 3–4 washes with PBS. The cells were then permeabilized using PBS supplemented with 0.1% Triton X-100 (*v*/*v*) for 30 min followed by blocking in blocking buffer (PBS supplemented with 2% bovine serum albumin [*w*/*v*]) for 1 h. After blocking, cells were treated with primary antibodies specific to NP in the blocking buffer. After 3–4 washes with PBS supplemented with 0.05% Triton X-100, the cells were incubated with DyLight488-labelled goat anti-mouse secondary antibodies (ThermoFisher Scientific^TM^, USA) (1:200) diluted in blocking buffer at room temperature for 1 h. After 3–4 washes with PBS supplemented with 0.05% Triton X-100, the cells were mounted with 4′, 6′-diamidino-2-phenylindole (DAPI) and covered with a coverslip to visualize the nuclei. Imaging was conducted using a Zeiss Axioplan LSM 710 microscope, 63×/N.A. 1.4 Oil immersion DICⅢ. Acquired images were analyzed using Zen Blue software. A Scattergram of co-localized pixels and Pearson’s correlation coefficient were generated using Zen Blue software (version 3.4.91.00000).

### 2.7. Plasmid Transfection

HEK293 cells were transfected with empty pcDNA3 vector or pcDNA3 vector carrying the insert for the IAV-HA gene (designated as pcDNA3-HA) using Lipofectamine™ 2000 Transfection Reagent (ThermoFisher Scientific) following the manufacturer’s protocol. For transfection, HEK293 cells were plated onto 6-well plates. An amount of 2 µg of each plasmid and Lipofectamine™ 2000 Transfection Reagent was incubated in Opti-MEM™ Reduced Serum Medium (Gibco, USA), followed by treatment of the cells in Opti-MEM™ media with the DNA–lipid complex for 24 h.

### 2.8. Nucleus–Cytosol Extraction

Nuclear and cytosolic fractions of IAV/PR8-infected cells and IAV/PR8-infected cells treated with minocycline or MEK/ERK pathway inhibitor PD98059 (30 µM) were separated and extracted using NE-PER™ Nuclear and Cytoplasmic Extraction Reagents (78833; Thermo Scientific™, St. Louis, MO, USA) following the manufacturer’s instructions. Briefly, the cell pellets were vortexed in ice-cold Cytoplasmic Extraction Reagent I and II and centrifuged at maximum speed. The obtained supernatant was kept as the cytoplasmic fraction and the insoluble pellet containing the nuclei was dissolved in an ice-cold Nuclear Extraction Reagent. The dissolved nuclear pellets were vortexed and finally centrifuged to obtain the supernatant, which was used as the nuclear protein fraction. The protein concentration of the fractions was determined using Bradford reagent (Sigma-Aldrich, USA). The fractions were run on SDS-PAGE followed by Western blotting using anti-NP antibody. Anti-β-Actin and anti-Lamin A/C antibodies were used for verifying the purity of cytosolic and nuclear fractions, respectively.

### 2.9. Cell Viability Assay 

Cells grown in 96-well plates with 80–90% cell confluency were treated with minocycline at different concentrations (1 µM–100 µM) for 3 days followed by an MTT assay. Briefly, 50 µL of each MTT solution prepared in PBS (5 mg/mL) and serum-free media were added to each well and incubated at 37 °C for 4 h. The yellow tetrazolium salt, i.e., 3-(4,5-dimethylthiazol-2-yl)-2,5-diphenyltetrazolium bromide is reduced to purple formazan crystals by the metabolically active cells. These formazan crystals were dissolved in 200 µL of DMSO and the optical density (OD) of the solution was measured at 570 nm to obtain the sample signal. The percentage of cell viability was measured using the following formula: (OD_Sample_ − OD_Blank_) × 100/(OD_Control_ − OD_Blank_).

### 2.10. Hemagglutination (HA) Assay

The cells were incubated at desired conditions and the supernatant was collected. An amount of 50 µL of PBS (pH 7.2) was added to each well, except the first column, of a labeled U-bottom 96-well microtiter plate. An amount of 100 µL of supernatant (antigen) was added to the first column and serially ½ diluted across each row until the 11th column. An amount of 50 µL of 1% chicken RBCs was added to each well and was left undisturbed at room temperature for 60 min. The RBCs will either form a ring/button or show agglutination (hemagglutination) at the bottom of the well. The highest dilution of the virus at which hemagglutination occurs is considered the end point of the titration. The reciprocal of this dilution of the virus gives the HA titer.

### 2.11. Apoptosis Assay

MDCK cells grown on glass coverslips (~70% confluency) were minocycline-treated and/or infected with IAV/PR8. Staurosporine-treated cells as an apoptosis inducer served as the positive control while mock-infected cells were treated as the negative control. After 24 h of infection, the supernatant was discarded and the cells were washed thrice with PBS and then allowed to equilibrate with 1X annexin buffer (V13241; Thermo Scientific™) for 2 min at 37 °C. The cells were stained with annexin V diluted in 1X annexin buffer and incubated at 37 °C for 20 min in the dark. As per the manufacturer’s protocol, cells on coverslips were fixed with 2% formaldehyde mounted with 100% glycerol and visualized under a Zeiss Axioplan LSM 710 microscope, 63×/N.A. 1.4 Oil immersion DICⅢ. Acquired images were analyzed using Zen Blue software (version 3.4.91.00000). A scattergram of co-localized pixels and Pearson’s correlation coefficient were generated using Zen Blue software. In apoptotic cells, the phosphatidylserine present in the inner leaflet of the lipid bilayer is exposed on the outer layer. Fluorophore FITC-conjugated Annexin V recognizes phosphatidylserine on the outer leaflet and binds with high affinity, thereby distinguishing apoptotic cells from non-apoptotic ones. The intensity of the fluorophore is measured and represented as intensity (%) when compared to mock-infected cells.

### 2.12. Plaque Assay

The cells were incubated at desired conditions and the supernatant containing IAV/PR8 was collected. An assay was conducted as per previous studies [39]. Briefly, MDCK cells grown on a 12-well plate (~90% confluency) were infected with a gradient dilution of the supernatant containing IAV/PR8 in viral growth medium (VGM) for 60 min at 37 °C with agitation after every 20 min. Virus inoculum was then discarded and 1.5 mL of overlay (1.5% CM cellulose:2X DMEM = 1:1 supplemented with 2.5 µg/mL TPCK-treated trypsin and 0.3% BSA) was added to each well and incubated at 37 °C for 72 h and observed for plaque formation. The cells were then fixed with 4% paraformaldehyde for 10 min at room temperature; then, the overlay was discarded and the cells were stained with 0.2% crystal violet. The number of plaques was counted and PFU/mL was calculated using the following formula: (no. of plaques × dilution factor)/volume of inoculum. 

### 2.13. Statistical Analysis

All the experiments were replicated at least thrice and the data were analyzed by one-way and two-way analysis of variance (ANOVA), followed by Tukey’s test or unpaired or multiple *t*-tests using GraphPad 8 and represented as mean ± standard deviation (SD) of three independent experimental replicates. A *p* value less than 0.05 was considered significant.

## 3. Results

### 3.1. Minocycline Treatment Showed Potent Anti-IAV Activity In Vitro

**Figure 1:**
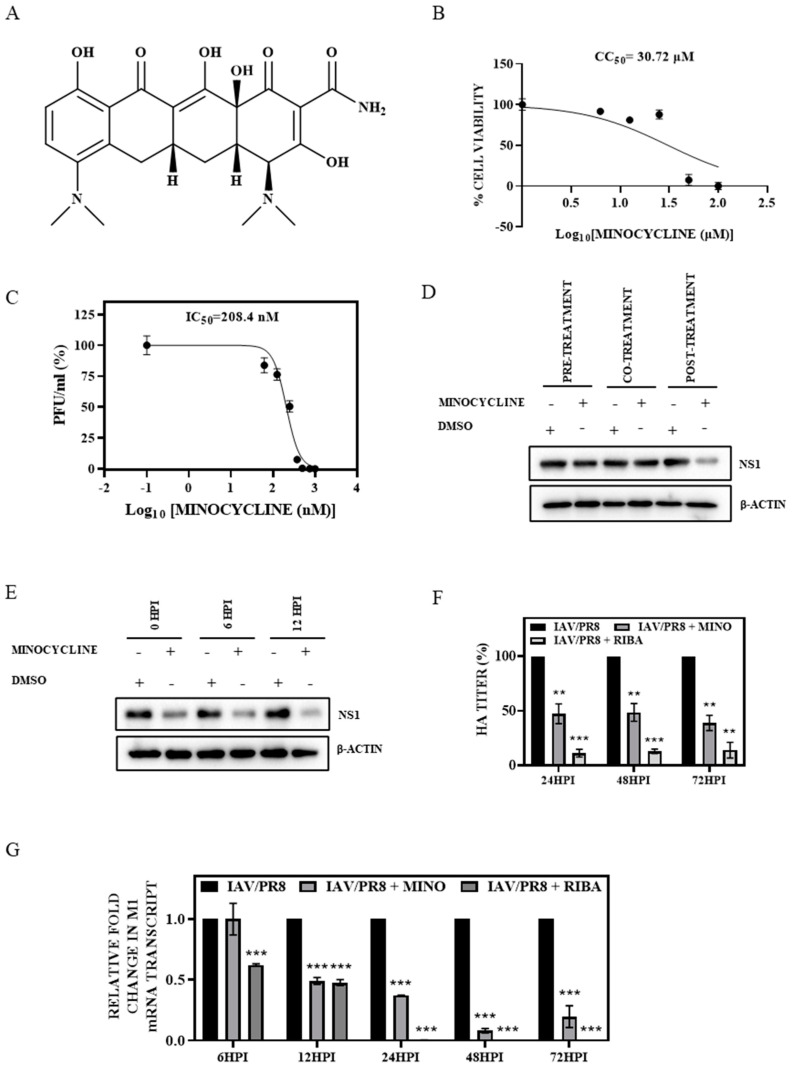
(**A**) Chemical structure of minocycline. (**B**) Cell viability of MDCK cells following treatment with different concentrations of minocycline (1 nM–100 µM) for 72 h was measured using the MTT assay to calculate CC_50_. (**C**) Infectious IAV particles produced from IAV/PR8 MDCK cells subjected to varying concentrations of minocycline (0.1 nM–1 µM) for 72 h were used to calculate IC_50_. (**D**) IAV/PR8-infected MDCK cells were treated with DMSO or minocycline (500 nM) 1 h prior to infection (pre-treatment), at the time of virus addition (co-treatment) and 1 h after viral addition (post-treatment) to assess its effect on viral protein expression. Western blotting was conducted after 24 h to check NS1 protein along with the loading control β-actin. (**E**) Post-treatment of DMSO or minocycline (500 nM) at 0 h, 6 hpi, and 12 hpi to IAV/PR8-infected cells to assess the stage of viral life cycle inhibited by minocycline. Western blotting was performed after 24 h to check NS1 protein along with the loading control β-actin. (**F**) Evaluation of HA titer from supernatant extracted from IAV/PR8-infected MDCK cells treated with DMSO or 500 nM of minocycline (at 24, 48, and 72 hpi); cells treated with 100 µM of ribavirin served as the positive control. A hemagglutination assay was performed to estimate viral titers. Each bar represents the mean value ± SD of three independent experiments (two-way ANOVA, ** *p* < 0.01, *** *p* < 0.001). (**G**) Relative expression of mRNA extracted from IAV/PR8-infected MDCK cells treated with DMSO or 500 nM of minocycline (at 6, 12, 24, 48, and 72 hpi) was estimated by quantitative RT-PCR. Cells treated with 100 µM of ribavirin served as the positive control. Each bar represents the mean value ± SD of three independent experiments (one-way ANOVA, *** *p* > 0.001).

To assess the cytotoxicity of minocycline, the effect of increasing the concentration of minocycline (structure shown in Figure 1A) on the viability of MDCK cells was evaluated over a 72 h period using the MTT assay. Compared to DMSO-treated control cells, a 50% cell death (CC_50_) was observed upon treatment with 30.72 µM minocycline (Figure 1B), signifying a relatively low cytotoxic effect of the drug. Subsequently, the antiviral potential of minocycline at non-toxic doses was assessed in MDCK cells infected with IAV/PR8. Virus-infected MDCK cells were subjected to increasing concentrations of minocycline (0.1 nM to 1 µM) for 72 h and cell supernatant was used to quantify the virus yield by plaque assay. A 50% reduction in viral titer (IC_50_) was noted at a non-toxic dose of 208.4 nM of minocycline. This confirmed a high Selectivity Index (SI = CC_50_/IC_50_) of 147.4 for minocycline against IAV infection (Figure 1C). Based on the CC_50_/IC_50_ data, a 500 nM dose of minocycline was used in further experiments, as more than 50% inhibitory effect on IAV production with negligible cytotoxicity was observed at this dose. To discern the stage of the viral life cycle impacted by minocycline treatment, infected cells were either pre-treated (1 h prior to infection), co-treated (during infection), or post-treated (at the time of virus removal). The results revealed that post-treatment of infected cells with minocycline exhibited maximum antiviral effect, while pre- and co-treatment had no significant impact on viral production and viral transcript synthesis (Figure 1D), signifying that minocycline does not influence viral adsorption or entry. A time-of-addition study was conducted to corroborate its effect on different stages of the viral life cycle. Administering minocycline at 0 hpi, 6 hpi, and 12 hpi followed by cell lysis at 24 hpi revealed substantial antiviral activity of minocycline when the drug was added at 6 hpi or 12 hpi. This suggests that minocycline modulates viral replication during the last stage of the life cycle (Figure 1E). To further elucidate the anti-IAV activity of minocycline, the temporal alterations in viral transcript synthesis and viral production were assessed following minocycline treatment. Expectedly, treatment of IAV/PR8-infected MDCK cells with 500 nM of minocycline resulted in over 50% reduction in HA titers at 24, 48, and 72 hpi and viral (M1) mRNA transcript synthesis at 12, 24, 48, and 72 hpi (Figure 1F,G). Similarly, a reduction in viral titer (PFU/mL) in the presence of minocycline was confirmed by plaque assay (Supplementary Materials Figure S4A). To rule out cell line-specific effects, the antiviral activity of minocycline was also confirmed in A549 cells infected with IAV/PR8 (Supplementary Materials Figure S2E). IAV/PR8-infected MDCK cells treated with 100 µM of ribavirin served as the positive control. In concordance with transcript synthesis, a gradual decline in IAV protein (NS1) expression was also observed following minocycline treatment (500nM) in MDCK cells infected with either cell culture-adapted strain IAV/PR8 or pandemic H1N1 virus strain IAV/CAL (Supplementary Materials Figure S1A,B). Reduction in HA titers was also observed in cells infected with IAV/CAL or IAV/H3N2 following treatment with minocycline (Supplementary Materials Figure S1C,D). Cumulatively, these findings underscore the anti-influenza activity of minocycline in vitro.

### 3.2. Minocycline Impedes IAV Infection without Triggering Interferon (IFN) Signaling

**Figure 2:**
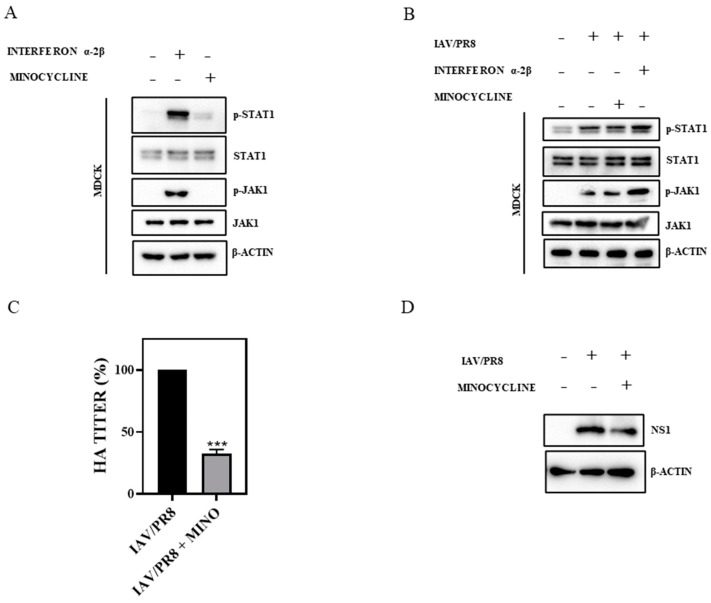
(**A**) MDCK cells were treated with minocycline (500 nM) for 12 h and a Western blot was performed to record any change in phosphorylation of JAK/STAT. IFN α-2β served as the positive control. (**B**) IAV/PR8-infected MDCK cells were treated with minocycline (500 nM) for 24 h and a Western blot was performed to record any change in phosphorylation of JAK/STAT. IFN α-2β served as the positive control. (**C**) IFN signaling-deficient Vero cells were infected with IAV/PR8 and treated with minocycline (500 nM). The HA titer from the supernatant was estimated by hemagglutination assay. Each bar represents the mean value ± SD of three independent experiments (unpaired *t*-test, *** *p* < 0.001). (**D**) A Western blot was performed to estimate the reduction in viral protein NS1, with β-actin serving as the loading control.

Type 1 IFNs, such as interferon α and interferon β, are known as critical components of the innate immune response, primarily to combat viral infections by principally targeting the JAK1-STAT1 pathway [40]. To investigate the potential of minocycline to induce the IFN signaling pathway, we assessed the activation status of the JAK1-STAT1 pathway in MDCK and A549 cells treated with 500 nM of minocycline for 12 h. In parallel, cells were treated with IFN α-2β as a positive control. In contrast to the robust phosphorylation of JAK1 and STAT1 in IFN α-2β-treated cells, minocycline failed to stimulate JAK1 and STAT1 phosphorylation (Figure 2A, Supplementary Materials Figure S2A), suggesting no effect of the drug on the IFN signaling pathway. Furthermore, in MDCK and A549 cells infected with IAV, minocycline did not alter the IAV-induced phosphorylation of JAK1 and STAT1 at 24 hpi (Figure 2B, Supplementary Materials Figure S2B). Additionally, the anti-IAV potential of minocycline was also evident in IFN signaling-deficient Vero cells, as demonstrated by reduced HA titers at 24 hpi (Figure 2C) and reduced synthesis of viral non-structural protein NS1 (Figure 2D). Collectively, these results confirm that the anti-IAV potential of minocycline is independent of IFN signaling.

### 3.3. Minocycline Prevents the Shuttling of vRNPs from the Nucleus to Cytosol by Inhibiting the ERK Pathway

**Figure 3:**
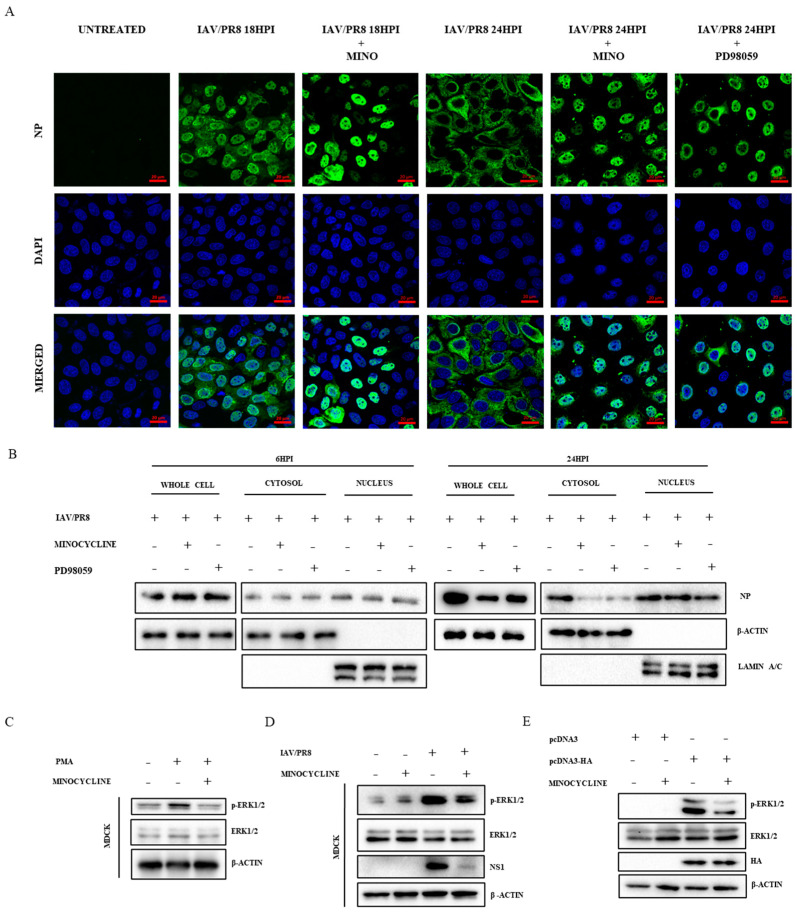
(**A**) IAV/PR8-infected MDCK cells treated with minocycline (500 nM) or PD98059 (30 µM) and incubated for 18 and 24 hpi. The cells were then fixed, permeabilized, and stained with anti-NP antibodies (raised in mice). The cells were then secondarily stained with DyLight488-labeled anti-mouse secondary antibody. The cells were mounted using DAPI and visualized under a confocal microscope (63X oil immersion). Scale bar: 20 µm. (**B**) Whole-cell, nuclear, and cytosolic fractions of IAV/PR8-infected cells treated with minocycline (500 nM) or PD98059 (30 µM) at 6 hpi and 24 hpi were extracted. A Western blot was performed with β-actin serving as the loading control for cytosolic and whole-cell lysate and lamin A/C for nuclear fractions. (**C**) Proteins from PMA-induced MDCK cells at 18 hpi were extracted and Western blot analysis was performed to observe phosphorylation of ERK, with β-actin serving as the loading control. (**D**) Western blot analysis of IAV/PR8-infected MDCK cells treated with minocycline (500 nM) was conducted to observe phosphorylation of ERK at 24 hpi. (**E**) HEK293T cells were transfected with only pcDNA3 vector and pcDNA3-HA plasmid and treated with 500 nM of minocycline for 24 h; the cells were lysed and a Western blot was performed to detect the level of phospho-ERK.

Nuclear import and export of vRNPs is the pivotal step in the life cycle of the influenza virus [41]. In this study, we examined the cellular localization of vRNPs visualized in IAV/PR8-infected MDCK cells treated with either DMSO or minocycline (500 nM) at 18 and 24 hpi by immunolabelling viral nucleoprotein NP, which is a key component of the vRNP complex. Immunofluorescence microscopy revealed that in IAV-infected cells treated with minocycline, NP protein accumulated within the nucleus with a concomitant decrease in overall IAV-infected cells compared to the DMSO treatment at both 18 and 24 hpi (Figure 3A, Supplementary Materials Figure S3C). Western blot analysis corroborated these findings, indicating an accumulation of NP protein in nuclear fractions relative to whole-cell lysate at 24 hpi, suggesting the role of minocycline in modulating nuclear export of vRNPs (Figure 3B, Supplementary Materials Figure S3A,B). Notably, despite the reduction in total viral NP protein at 24 hpi, three times more NP protein was observed to be localized in the nucleus than in the cytosol. This result is consistent with its antiviral effect during the later stage of infection. Previous studies have indicated that the nuclear export of vRNPs is regulated by the cellular kinase ERK [42,43]. Consistently, immunofluorescence and Western blot data also showed nuclear accumulation of NP protein following treatment with ERK inhibitor PD98059 (30 µM) (Figure 3A,B, Supplementary Materials Figure S3A,B). These findings led us to hypothesize that minocycline may regulate ERK. To test this hypothesis, cells were treated with the ERK inducer PMA in the presence or absence of minocycline for 18 h and the phosphorylation status of ERK1/2 was evaluated by Western blot analysis. As shown in Figure 3C and Supplementary Materials Figure S2C, minocycline completely abolished the PMA-induced phosphorylation of ERK1/2 without affecting the basal level expression in both MDCK and A549 cells. IAV-induced ERK1/2 phosphorylation was also found to be reduced upon treatment with minocycline at 24 hpi in both MDCK and A549 cells (Figure 3D, Supplementary Materials Figure S2D). Transient expression of IAV protein HA has previously been shown to induce ERK activation [44]. Therefore, we further assessed the inhibition of ERK by minocycline in HEK293T cells transiently expressing HA protein. Consistent with previous findings, minocycline treatment also reduced HA-induced phosphorylation of ERK in HEK293T cells (Figure 3E), thus confirming the inhibitory role of minocycline on ERK signaling.

### 3.4. Minocycline Impedes IAV-Induced Late-Stage Apoptosis

**Figure 4:**
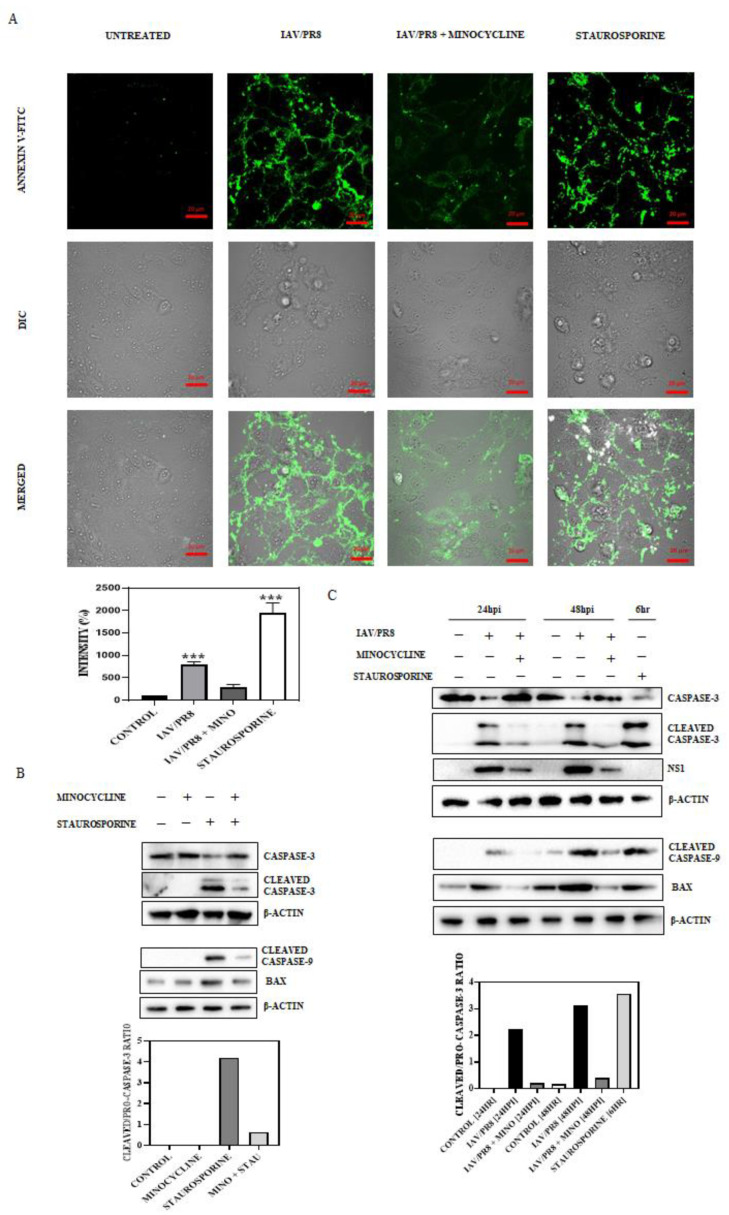
(**A**) IAV/PR8-infected MDCK cells were treated with minocycline (500 nM) for 24 hpi, after which the cells were stained with FITC-conjugated Annexin V and fixed. The cells were then mounted and observed under a confocal microscope (63× oil immersion) and the intensity of FITC was quantified and graphically represented. Staurosporine-treated cells were treated as the positive control. Each bar represents the mean value ± SD of three independent experiments (one-way ANOVA, *** *p* < 0.001). (**B**,**C**) MDCK cells treated for 6 h with minocycline or staurosporine, or a combination of both, were tested for bax and cleavage of caspase-3 and caspase-9. Western blotting was conducted to quantify the intensity of bax, pro-caspase-3, cleaved caspase-3, and cleaved caspase-9 blots. The ratio of cleaved caspase-3 to pro-caspase-3 was deduced and plotted. In the case of IAV/PR8-infected MDCK cells treated with minocycline (500 nM) at 24 and 48 hpi, a similar estimation was conducted and plotted. Staurosporine-treated cells were treated as the positive control.

Induction of apoptosis during the late stage of the IAV life cycle has been implicated in viral progeny release and dissemination [45]. Minocycline was previously reported to inhibit apoptosis [17,46,47,48]. Therefore, to verify the anti-apoptotic role of minocycline, MDCK cells were treated with the apoptosis inducer staurosporine, both in the presence and absence of minocycline, for 6 h. A transcriptomic analysis in 2014 revealed upregulation of retinoic acid-mediated apoptosis by all IAV subtypes except for H7N9 [49]. Therefore, caspase-3 or caspase-9 cleavage, a key marker of apoptosis, was assessed. Bax, a pro-apoptotic protein, was also assessed upon minocycline treatment to staurosporine-induced or IAV-infected cells. Consistent with previous reports, minocycline treatment significantly inhibited staurosporine-induced caspase-3 or -9 cleavages and reduced levels of bax (Figure 4B). Inhibition of caspase-3 or -9 cleavages and reduced levels of bax was also confirmed in minocycline-treated IAV-infected cells compared to DMSO treatment at both 24 hpi and 48 hpi (Figure 4C). The ratio of cleaved caspase-3 to pro-caspase-3 indicated a marked reduction in caspase activation in both IAV-infected cells or staurosporine-induced cells treated with minocycline at 24 and 48 hpi. Labeling of apoptotic cells with Annexin V-FITC also revealed a significant reduction in the number of apoptotic cells in IAV-infected cells treated with minocycline (Figure 4A). Overall, these findings underscore that IAV-induced late-stage apoptosis is attenuated by minocycline, which possibly interferes with the progeny virus release and dissemination. 

### 3.5. Minocycline Treatment Attenuates IAV Infection In Vivo

**Figure 5:**
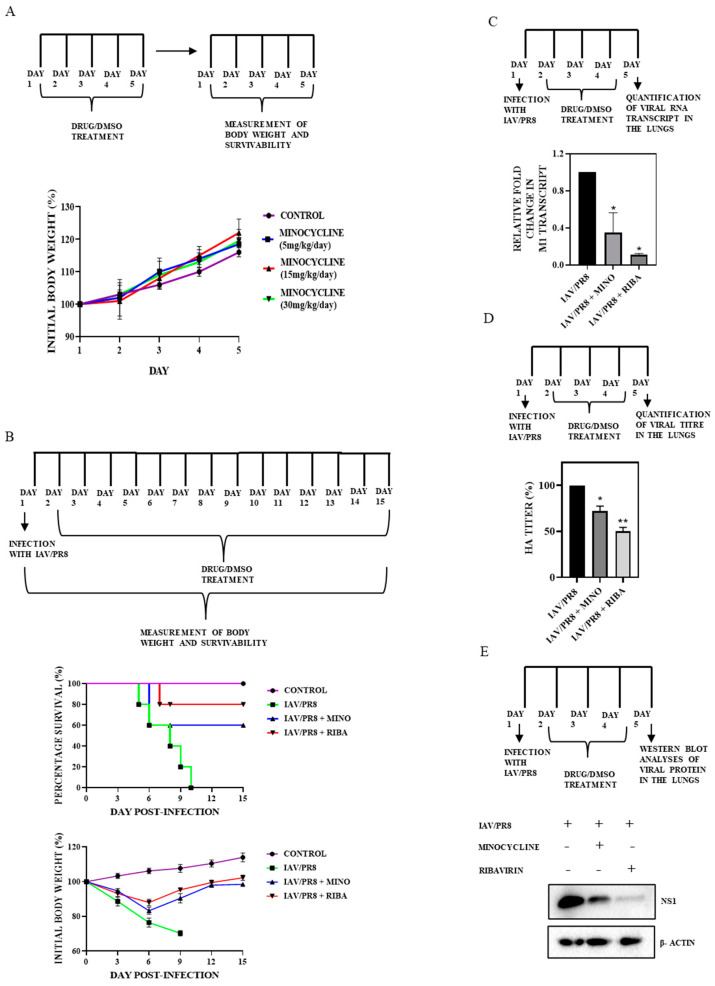
BALB/c mice (n = 5 mice/group) were orally treated with 5, 15, and 30 mg/kg/day of minocycline for 5 d to test its toxicity. (**A**) Body weight was measured daily for 5 d. (**B**) Female BALB/c mice (n = 5 mice/group) were intranasally infected with the IAV/PR8 (4 × 50% mouse lethal dose, MLD50). After 1 day, mice were treated with 30 mg/kg/day of minocycline or DMSO for 15 d. Body weight and survivability were measured daily for 15 d. (**C**) Female BALB/c mice (n = 5 mice/group) were intranasally infected with IAV/PR8 and treated with 30 mg/kg/day of minocycline or DMSO for 5 d. The mice were sacrificed and the lungs were excised and homogenized. Relative viral RNA levels in lungs at 5 dpi were determined by RT-PCR. Each bar represents the mean value ± SD of three independent experiments (one-way ANOVA, * *p* < 0.05). (**D**) Relative HA titers in the lungs were measured by hemagglutination assay. Each bar represents the mean value ± SD of three independent experiments (one-way ANOVA, * *p* < 0.05, ** *p* < 0.001). (**E**) Viral protein NS1 level was estimated by Western blotting. Mice treated with ribavirin (70 mg/kg/day) served as the positive control.

**Figure 6:**
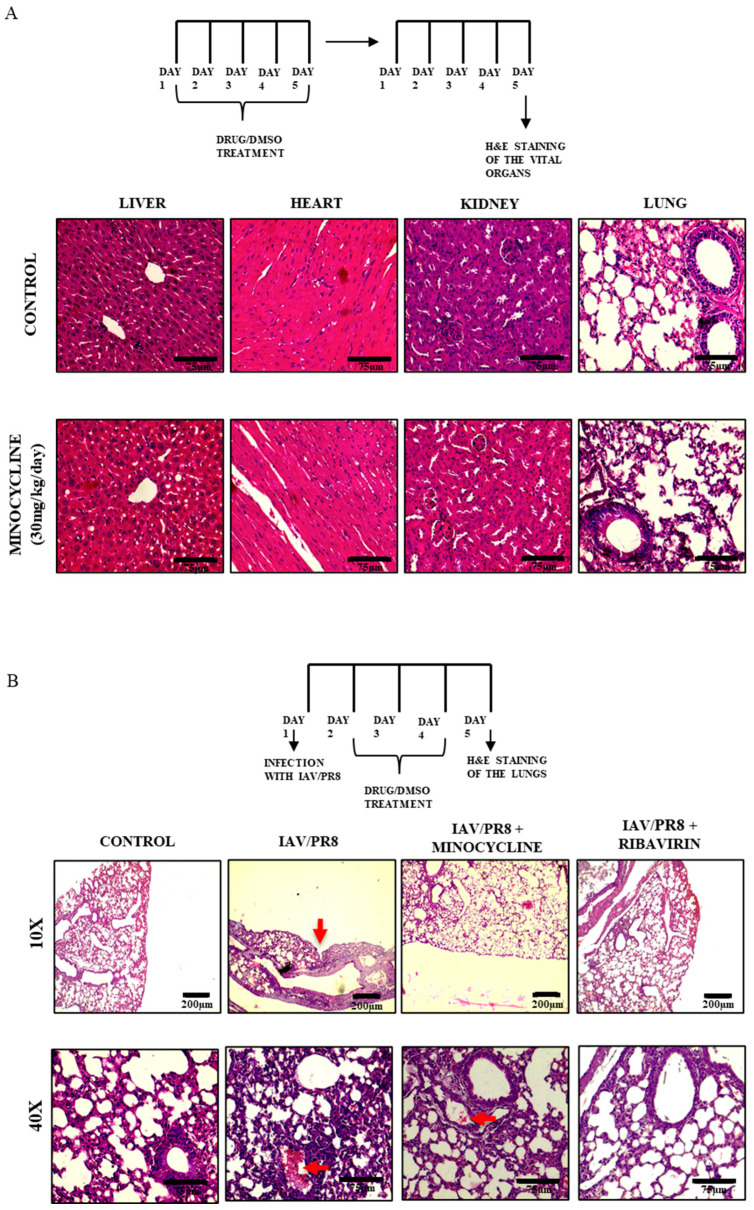
(**A**) BALB/c mice (n = 5 mice/group) were orally treated with 5, 15, and 30 mg/kg/day of minocycline for 5 d to test its toxicity. H&E staining of the vital organs was carried out on day 5 and observed under 40× magnification, with a scale bar of 75 µm. (**B**) BALB/c mice (n = 5 mice/group) were intranasally infected with IAV/PR8 and treated with 30 mg/kg/day of minocycline or DMSO for 5 d. The mice were sacrificed and the lungs were excised and homogenized. H&E staining of the lung tissues was conducted and observed under 10× and 40× magnification, with scale bars of 75 µm and 200 µm, respectively. Changes in lung morphology are indicated with red arrows.

To further confirm the anti-IAV activity of minocycline, its role was assessed in a mouse model of IAV infection. Initially, to standardize the dose of minocycline, female BALB/c mice (4–6 weeks, n = 5) were orally administered with increasing doses of minocycline (5–30 mg/kg/day) or vehicle control for 5 consecutive days and their body weight was monitored daily. Notably, no significant changes in body weight were observed even after 5 days of consecutive treatment with the highest dose (30 mg/kg/day) of minocycline compared to the vehicle control (DMSO) (Figure 5A), suggesting that dosage up to 30 mg/kg/day is well tolerated. Histological examination with H&E staining of major vital organs such as the liver, heart, kidney, and lungs revealed no discernible impact on tissue morphology in mice treated with minocycline (30 mg/kg/day) (Figure 6A). 

Subsequently, the antiviral effect of minocycline against IAV/PR8 was assessed. The mice (n = 5) were intranasally inoculated with IAV/PR8 on day 1, and from the subsequent day until day 15 they were orally administered with minocycline (30 mg/kg/day). During this period, their body weight and survival rates were monitored. As depicted in Figure 5B, minocycline conferred a protective effect against IAV/PR8 infection, with a higher survival rate of 67% compared to vehicle-treated infected mice. None of the vehicle-treated infected mice survived beyond 10 days. The body weight of IAV-infected and vehicle-treated mice experienced a significant decline, dropping to nearly 75% of their initial body weight by days 6–9. In contrast, IAV-infected mice treated with either minocycline or ribavirin demonstrated a reduction in body weight until day 6, followed by a steady increase in body weight thereafter (Figure 5B). 

To further substantiate the anti-IAV potential of minocycline, viral load was measured in the lungs of infected mice on day 5 post-infection. Significantly lower titers were detected in the lungs of IAV/PR8-infected mice treated with minocycline compared to DMSO-treated controls (Figure 5D, Supplementary Materials Figure S4B). Additionally, inhibition of IAV infection following treatment with minocycline for 5 days was also confirmed by the reduced expression of both IAV transcript M1 and NS1 protein (Figure 5C,E) in the lung tissue of IAV-infected mice treated with minocycline compared to those treated with DMSO. Consistently, H&E staining revealed reduced lesions indicative of influenza-induced lung pathology in minocycline-treated mice compared to vehicle-treated controls. Notably, the control group of mice exhibited considerable invagination of the pulmonary pleura. Moreover, thickening of the pulmonary septum along with infiltration of inflammatory cells (indicated with red arrows) were observed in IAV/PR8-infected mice; these effects were evidently reduced in minocycline-treated mice (Figure 6B) [50].

## 4. Discussion

An intrinsic feature of accumulation of mutations in the influenza virus poses significant challenges towards developing effective antiviral drugs targeting virus-encoded proteins. The mutations in the epitopes and the evolution of genetic reassortant viruses render the use of antivirals, such as NA inhibitors and M2-ion channel blockers, ineffective [51,52,53,54]. Overuse of these antivirals has led to the development of drug-resistant IAV strains, prompting the need to seek alternative therapeutic strategies, including drug repurposing. This approach involves assessing the antiviral potential of previously approved drugs, including antimicrobials, anticancer drugs, and phytochemicals [55,56,57,58]. The tetracycline derivative known as minocycline, which was synthesized in 1967, has demonstrated efficacy in the management of infections, including rickettsial infection, syphilis, acne, etc. [59]. The Infectious Diseases Society of America (IDSA) endorsed minocycline for treating infections caused by carbapenem-resistant *Acinetobacter baumannii* and *Stenotrophomonas maltophilia* in 2022 [60]. Few reports show no improvement in disease conditions in the case of flu patients treated with minocycline; however, such results may vary due to different geographical locations, disease conditions, and susceptibility of the patient [61].

**Figure 7:**
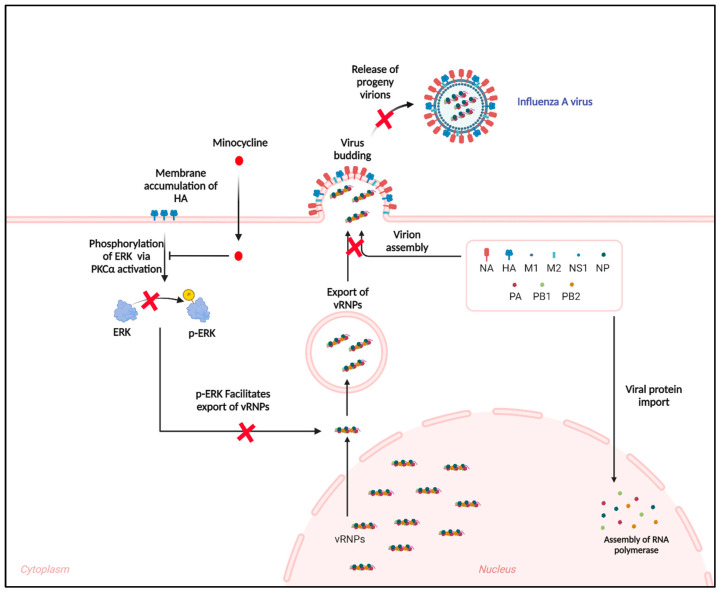
A hypothetical scheme of the role of minocycline in hindering the export of vRNPs from the nucleus leading to inhibition of viral assembly and dissemination.

Minocycline also exhibited antiviral potential against the West Nile Virus (WNV). Minocycline inhibits WNV-induced apoptosis by downregulating the JNK cascade [26]. It also influences viral replication by activating CD4+ T cells in HIV-infected patients [29]. Its anti-apoptotic nature was also established in the context of Japanese encephalitis virus (JEV)-induced microglial activation in neuronal cells [27]. A computational and in vitro study in 2014 showed that minocycline was capable of inhibiting replication of IAV H7N9 but its mechanism remained unexplored [49]. In contrast, a report by Inoue et al. reported no improvement in a pneumonia patient following minocycline treatment. However, in this case, minocycline was administered on day 6 of the illness, and the patient tested positive for *Streptococcus pneumoniae*, suggesting secondary bacterial infection [61]. The current study highlights that minocycline exhibits antiviral activity against IAVs in both in vitro and in vivo mice models. This antiviral activity was independent of IAV strains (IAV/H1N1, IAV/CAL, and IAV/H3N2) or cell lines (MDCK, A549, and Vero). Notably, minocycline has no direct effect on viral entry or transcription but inhibits the nuclear export of vRNPs. Viruses including IAVs exploit the mitogen-activated protein kinase (MAPK) signaling cascade, particularly the phosphorylation of the kinase Raf via MEK to ERK [62,63,64,65,66,67,68,69]. IAVs replicate within the nucleus and translate and assemble within the cytosol. Upon translation, the viral proteins (PA, PB1, PB2, and NP) are shuttled into the nucleus to interact with the newly synthesized vRNAs to form vRNPs, which are then exported from the nucleus for viral assembly and dissemination. Nuclear export protein (NEP) is reported to play a role in the nuclear export of vRNPs by interacting with cellular nuclear export protein Crm1 and viral Matrix protein 1 (M1) bound to vRNPs. It acts as an adaptor, thereby mediating vRNP export from the nucleus for packaging into progeny viruses [70]. Several cellular factors have been reported to contribute to this process. Previous studies have shown that the accumulation of HA protein in the membrane triggers phosphorylation of ERK1/2 via protein kinase Cα, promoting vRNP nuclear export [71]. Although NEP is not a direct target of mitogen-activated protein kinase (MAPK) signaling, inhibition of MAPK signaling interferes with the NEP-mediated vRNP export, though its mechanism is unknown [42]. Minocycline effectively inhibited phosphorylation of ERK, even in HA-overexpressing cells, thus restricting the nuclear export of the vRNPs (Figure 7). As propagation of IAVs is governed via the Raf/MEK/ERK cascade, its antagonist, PD9089, also exhibited interruption of vRNP export. 

Apoptosis has been considered a defense strategy of the host against the invading virus [72]. Sadly, viruses have evolved to sabotage the host’s strategy in this battle between the host and the virus and utilize apoptosis for its benefit [73,74]. Therefore, it has positive as well as negative impacts on the IAV life cycle [75]. Early induction of apoptosis impedes viral replication, thereby acting in favor of the host, while its late induction facilitates viral release and dissemination of progeny virions [76]. Therefore, IAVs promote apoptosis in the later stage of the life cycle, which facilitates the liberation of progeny virions [45,66,77]. Anti-apoptotic and anti-inflammatory potential of minocycline has already been established [78]. Its anti-apoptotic role was observed in JEV infection [28]. Staurosporine (apoptosis inducer) and IAV-induced apoptosis were also found to be inhibited by minocycline, thereby hindering the liberation of progeny virions from the infected cells, as elucidated in this study. Overall, this study highlights the antiviral property of minocycline against IAVs as a cumulative effect of inhibition of apoptosis and restriction of nuclear export of vRNPs. There may be more direct or indirect effects of minocycline on virus replication that require further in-depth studies. However, the development of resistance against minocycline also is a concern. Therefore, based on a confirmed diagnostic test for influenza and the status of the patient, antibiotics like minocycline should be used along with the current antivirals.

## Data Availability

Data is available at https://figshare.com/s/648cfa4dadb5efe495df (accessed on 12 August 2024).

## Supplementary Materials

The following supporting information can be downloaded at https://www.mdpi.com/article/10.3390/v16081317/s1: Figure S1: (A,B) IAV/PR8- and IAV/CAL-infected MDCK cells were treated with different concentrations of minocycline (100 nM–1 µM) and viral protein was estimated by Western blotting after 24 h. (C,D) IAV/CAL- or IAV/H3N2-infected MDCK cells were treated with minocycline (500 nM) or ribavirin (100 µM) for 24, 48, and 72 hpi; the cell supernatant was collected and HA titers were estimated by hemagglutination assay. Each bar represents the mean value ± SD of three independent experiments (two-way ANOVA, * *p* < 0.05, ** *p* < 0.01, and *** *p* < 0.001); Figure S2: (A) A549 cells were treated with IFN α-2β or minocycline for 12 h and Western blotting was conducted to study the JAK1-STAT1 signaling pathway. (B) IAV/PR8-infected A549 cells were treated with minocycline (500 nM) for 24 h and a Western blot was performed to record any change in phosphorylation of JAK1/STAT1. IFN α-2β served as the positive control. (C) Proteins from PMA-induced A549 cells at 18 hpi were extracted and Western blot analysis was performed to observe phosphorylation of ERK, with β-actin serving as the loading control. (D) Western blot analysis of IAV/PR8-infected A549 cells treated with minocycline (500 nM) was carried out to observe phosphorylation of ERK at 24 hpi. (E) IAV/PR8-infected A549 cells were treated with minocycline (500 nM) or ribavirin (100 µM) for 24, 48, and 72 hpi; the cell supernatant was collected and HA titers were estimated by hemagglutination assay. Each bar represents the mean value ± SD of three independent experiments (two-way ANOVA, ** *p* < 0.01 and *** *p* < 0.001); Figure S3: (A,B) Whole-cell, nuclear, and cytosolic fractions of IAV/PR8-infected minocycline (500 nM) treated cells or IAV/PR8-infected cells treated with PD98059 (30 µM) at 6 hpi and 24 hpi were extracted. Western blotting was performed, with β-actin serving as the loading control for cytosolic and whole-cell lysate and lamin A/C for nuclear fractions. Densitometric analysis of blots for NP, Lamin, and β-actin was conducted and represented as the fold change in total NP protein and the fold change in the nuclear/cytosol ratio of NP protein. (C) IAV/PR8-infected MDCK cells were treated with minocycline (500 nM) or PD98059 (30 µM) and incubated for 18 and 24 hpi. The cells were fixed, permeabilized, and primarily stained with anti-NP antibody (raised in mice), followed by secondary staining with DyLight488-labeled anti-mouse secondary antibody. The cells were then mounted using DAPI and visualized under a confocal microscope (63X oil immersion). NP-positive cells were quantified and 100 cells from different fields were randomly selected and analyzed. Data were represented as a percentage of infected cells. Each bar represents the mean value ± SD of three independent experiments (one-way ANOVA, * *p* < 0.05); Figure S4: (A) IAV/PR8-infected MDCK cells were treated with minocycline (500 nM) or ribavirin (100 µM) for 24, 48, and 72 hpi; the cell supernatant was collected and viral titers were estimated by plaque assay and represented as PFU/mL. Each point represents the mean value ± SD of three independent experiments (multiple *t*-tests, * *p* < 0.05 and ** *p* < 0.01). (B) Female BALB/c mice (n = 3 mice/group) were intranasally infected with IAV/PR8 and treated with 30 mg/kg/day of minocycline or DMSO for 5 d. The mice were sacrificed and the lungs were excised and homogenized. Relative viral titers in the lungs were measured by plaque assay in terms of PFU/mL. Each point represents mean value ± SD (one-way ANOVA, ** *p* < 0.01).
