# Correction: Saha et al. Unveiling the Antiviral Potential of Minocycline: Modulation of Nuclear Export of Viral Ribonuclear Proteins during Influenza Virus Infection. *Viruses* 2024, *16*, 1317

**DOI:** 10.3390/v17030396

**Published:** 2025-03-11

**Authors:** Priyanka Saha, Ritubrita Saha, Ratul Datta Chaudhuri, Rakesh Sarkar, Mehuli Sarkar, Hemanta Koley, Mamta Chawla-Sarkar

**Affiliations:** 1 Division of Virology, ICMR-National Institute of Cholera and Enteric Diseases, Kolkata 700010, India; prishah24@gmail.com (P.S.); saharitubrita@gmail.com (R.S.); dc.ratul@gmail.com (R.D.C.); rakeshsarkar133@gmail.com (R.S.); mehulisarkar@gmail.com (M.S.); 2 Division of Bacteriology, ICMR-National Institute of Cholera and Enteric Diseases, Kolkata 700010, India; hemantakoley@hotmail.com

## Error in Figure 4A Label

In the original publication [1], there was a mislabeling in “Figure 4A”. The label for “Figure 4A” was incorrectly listed as [“STAUROSPORINE, IAV/PR8 + MINOCYCLINE, IAV/PR8, UNTREATED”]. The correct label should read [“UNTREATED, IAV/PR8, IAV/PR8 + MINOCYCLINE, STAUROSPORINE”]. This error occurred due to delinquency during the manuscript editing process. The figure with the corrected labels is mentioned below. The authors confirm that the scientific conclusions remain unchanged. This correction has been approved by the Academic Editor, and the original publication has been modified accordingly.

## Corrected Figure 4A

**Figure 4:**
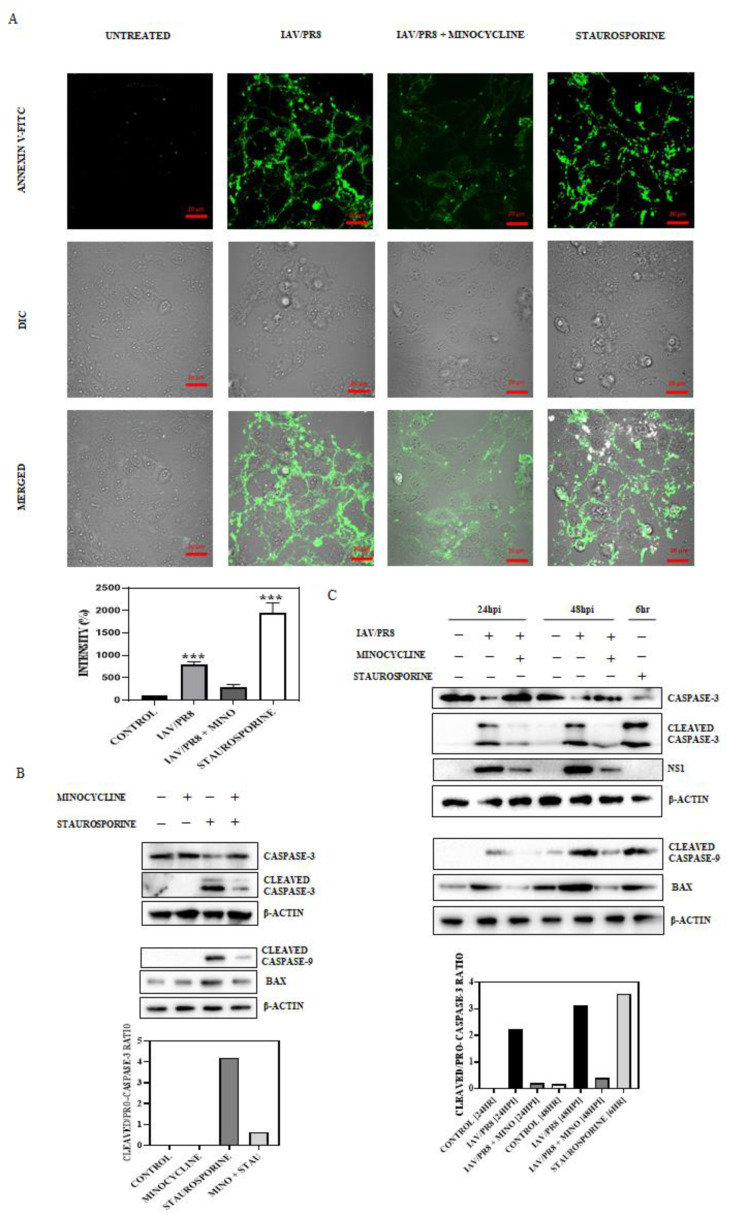
(**A**) IAV/PR8-infected MDCK cells were treated with minocycline (500 nM) for 24 hpi, after which the cells were stained with FITC-conjugated Annexin V and fixed. The cells were then mounted and observed under a confocal microscope (63× oil immersion) and the intensity of FITC was quantified and graphically represented. Staurosporine-treated cells were treated as the positive control. Each bar represents the mean value ± SD of three independent experiments (one-way ANOVA, *** *p* < 0.001). (**B**,**C**) MDCK cells treated for 6 h with minocycline or staurosporine, or a combination of both, were tested for bax and cleavage of caspase-3 and caspase-9. Western blotting was conducted to quantify the intensity of bax, pro-caspase-3, cleaved caspase-3, and cleaved caspase-9 blots. The ratio of cleaved caspase-3 to pro-caspase-3 was deduced and plotted. In the case of IAV/PR8-infected MDCK cells treated with minocycline (500 nM) at 24 and 48 hpi, a similar estimation was conducted and plotted. Staurosporine-treated cells were treated as the positive control.
