# Supplementary material for: Unveiling the Antiviral Potential of Minocycline: Modulation of Nuclear Export of Viral Ribonuclear Proteins during Influenza Virus Infection
Source: Viruses. 2024 Aug 18;16(8):1317. doi: 10.3390/v16081317 (PMC11359333; doi:10.3390/v16081317)
Supplement: Supplementary file 1 [file viruses-16-01317-s001.zip › viruses-3126396-supplementary.pdf]

viruses

MDPI

Supplementary Materials

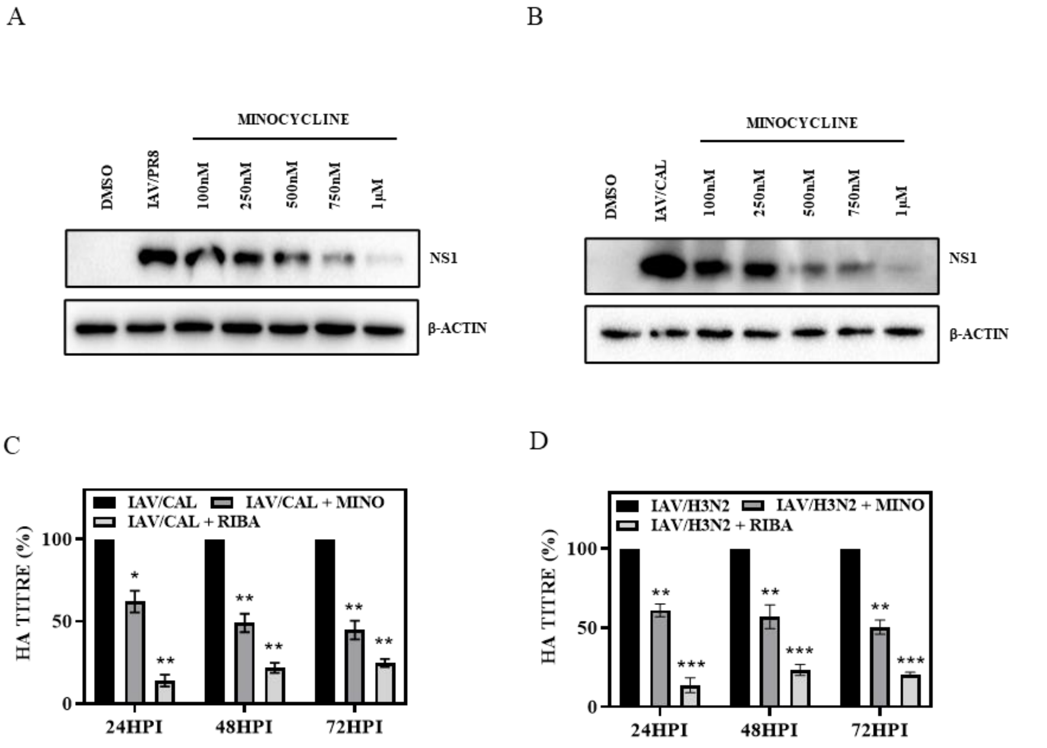

Supplementary Figure S1. (A,B) IAV/PR8- and IAV/CAL-infected MDCK cells were treated with different concentrations of minocycline (100 nM–1 μM) and viral protein was estimated by Western blotting after 24 h. (C,D) IAV/CAL- or IAV/H3N2-infected MDCK cells were treated with minocycline (500 nM) or ribavirin (100 μM) for 24, 48, and 72 hpi; the cell supernatant was collected and HA titers were estimated by hemagglutination assay. Each bar represents the mean value ± SD of three independent experiments (two-way ANOVA, * $p < 0.05$, ** $p < 0.01$, and *** $p < 0.001$).

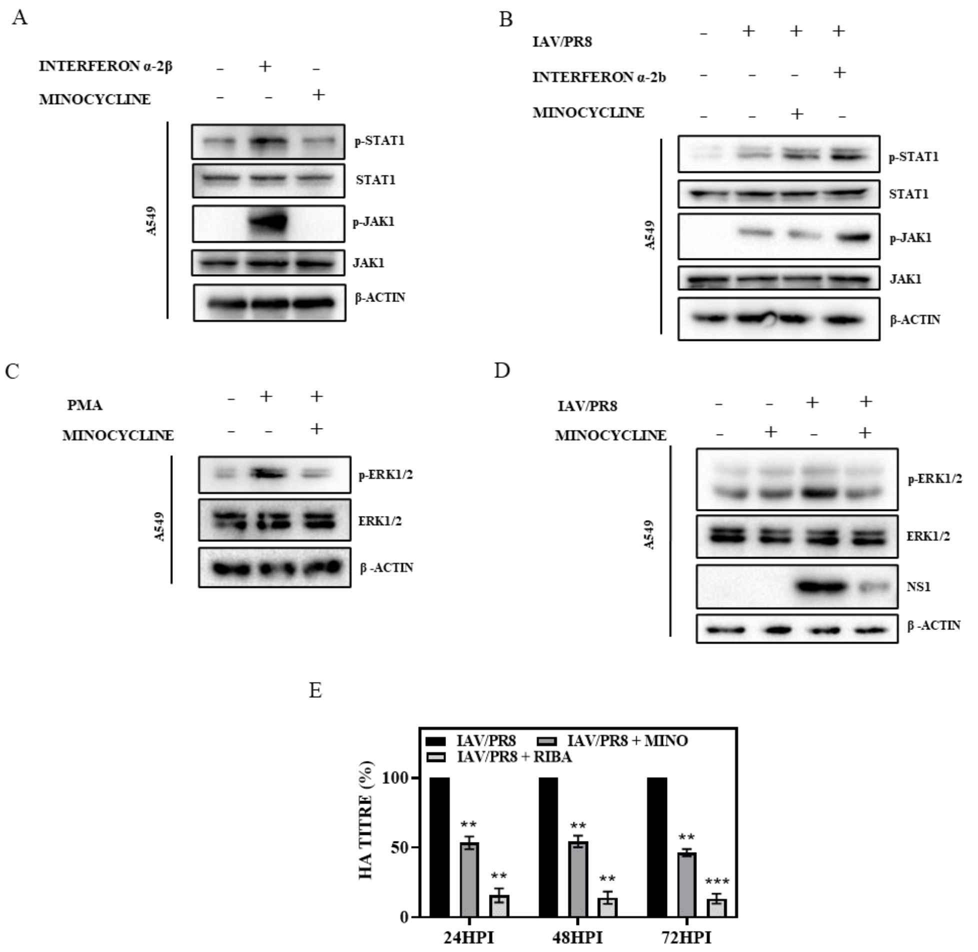

Supplementary Figure S2. (A) A549 cells were treated with IFN α-2β or minocycline for 12 h and Western blotting was conducted to study the JAK1-STAT1 signaling pathway. (B) IAV/PR8-infected A549 cells were treated with minocycline (500 nM) for 24 h and a Western blot was performed to record any change in phosphorylation of JAK1/STAT1. IFN α-2β served as the positive control. (C) Proteins from PMA-induced A549 cells at 18 hpi were extracted and Western blot analysis was performed to observe phosphorylation of ERK, with β-actin serving as the loading control. (D) Western blot analysis of IAV/PR8-infected A549 cells treated with minocycline (500 nM) was carried out to observe phosphorylation of ERK at 24 hpi. (E) IAV/PR8-infected A549 cells were treated with minocycline (500 nM) or ribavirin (100 μM) for 24, 48, and 72 hpi; the cell supernatant was collected and HA titers were estimated by hemagglutination assay. Each bar represents the mean value ± SD of three independent experiments (two-way ANOVA, ** $p < 0.01$ and *** $p < 0.001$).

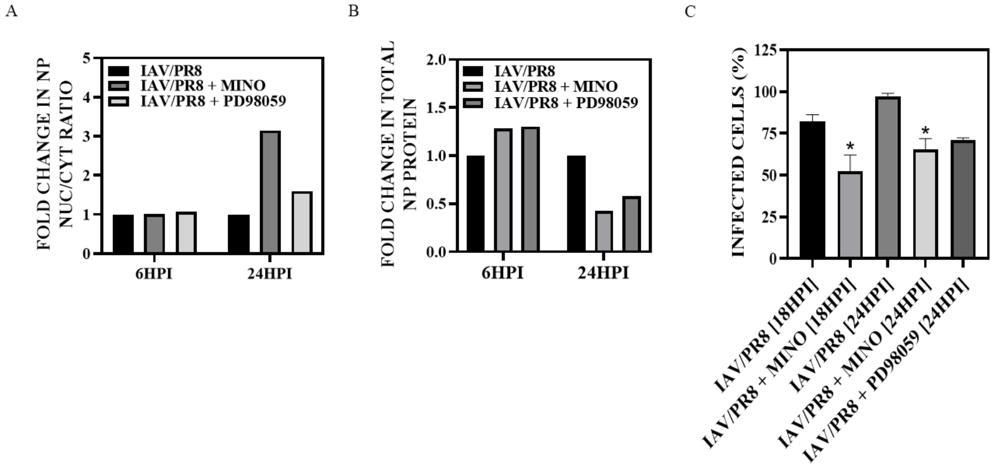

Supplementary Figure S3. (A,B) Whole-cell, nuclear, and cytosolic fractions of IAV/PR8-infected minocycline (500 nM) cells or cells treated with PD98059 (30 μM) at 6 hpi and 24 hpi were extracted. Western blotting was performed, with β-actin serving as the loading control for cytosolic and whole-cell lysate and lamin A/C for nuclear fractions. Densitometric analysis of blots for NP, Lamin, and β-actin was conducted and represented as the fold change in total NP protein and the fold change in the nuclear/cytosol ratio of NP protein. (C) IAV/PR8-infected MDCK cells were treated with minocycline (500 nM) or PD98059 (30 μM) and incubated for 18 and 24 hpi. The cells were fixed, permeabilized, and primarily stained with anti-NP antibody (raised in mice), followed by secondary staining with DyLight488-labeled anti-mouse secondary antibody. The cells were then mounted using DAPI and visualized under a confocal microscope (63X oil immersion). NP-positive cells were quantified and 100 cells from different fields were randomly selected and analyzed. Data were represented as a percentage of infected cells. Each bar represents the mean value ± SD of three independent experiments (one-way ANOVA, * $p < 0.05$).

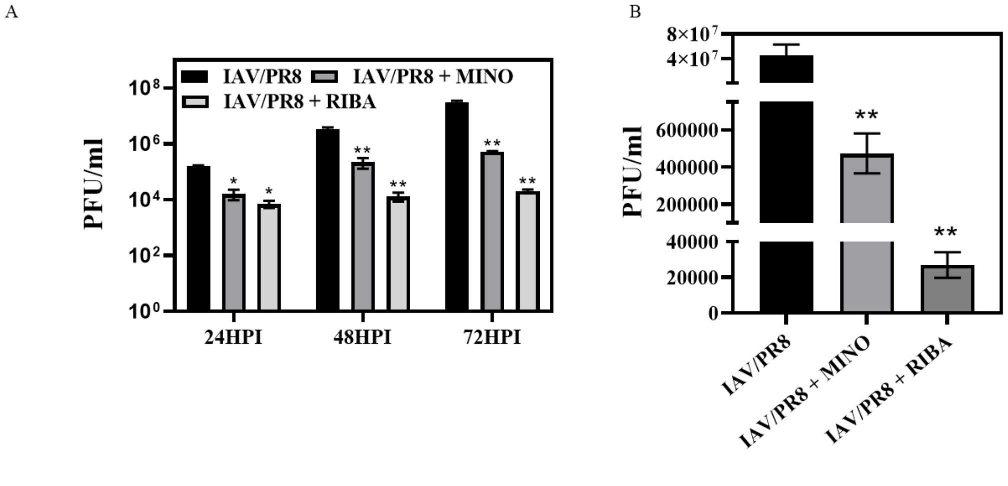

Supplementary Figure S4. (A) IAV/PR8-infected MDCK cells were treated with minocycline (500 nM) or ribavirin (100 μM) for 24, 48, and 72 hpi; the cell supernatant was collected and viral titers were estimated by plaque assay and represented as PFU/mL. Each point represents the mean value ± SD of three independent experiments (multiple *t*-tests, * $p < 0.05$ and ** $p < 0.01$). (B) Female BALB/c mice (n = 3 mice/group) were intranasally infected with IAV/PR8 and treated with 30 mg/kg/day of minocycline or DMSO for 5 d. The mice were sacrificed and the lungs were excised and homogenized. Relative viral titers in the lungs were measured by plaque assay in terms of PFU/mL. Each point represents mean value ± SD (one-way ANOVA, ** $p < 0.01$).
